# Numerical solution for circular tunnel excavated in strain-softening rock masses considering damaged zone

**DOI:** 10.1038/s41598-022-08531-3

**Published:** 2022-03-16

**Authors:** Jinwang Li, Caihua Shen, Xiufeng He, Xiangtian Zheng, Jiaojiao Yuan

**Affiliations:** 1 School of Earth Sciences and Engineering, Hohai University, Nanjing, 211100 China; 2 School of Civil and Transportation Engineering, Hohai University, Nanjing, 210098 China; 3 School of Computer Engineering, Nanjing Institute of Technology, Nanjing, 211167 China; 4 School of Construction Engineering, Jiangsu Open University, Nanjing, 210036 China

**Keywords:** Geology, Petrology, Sustainability, Engineering, Civil engineering

## Abstract

Despite the extensive investigation on the stress and displacement distributions in tunnels, few have considered the influences of the damaged zone around a tunnel on the strength and stiffness parameters of the surrounding rock, including the gradual variation in the damaged factor *D* and dimensionless damaged radius $\rho^{{\text{d}}}$, under the effect of excavation disturbance. In this paper, a numerical solution is presented for the stresses and displacement of a circular tunnel excavated in a Hoek–Brown rock mass considering the progressive destruction of the damaged zone. First, the results obtained using the proposed algorithm are compared with those obtained using other numerical solutions, demonstrating a high degree of accuracy through some examples. Second, the influences of the damaged factor $D$ and dimensionless damaged radius $\rho^{d}$ on the stresses, radial displacement, plastic radii, and ground response curve are investigated. The results show that, as the damaged factor *D* increases, the radial displacement and plastic radius increase, whereas the tangential stress decreases. Both the plastic radius and displacement decrease with decreasing $\rho^{{\text{d}}}$. This shows that the damaged factor *D* has a significant effect on tunnel convergence.

## Introduction

**Figure 1:**
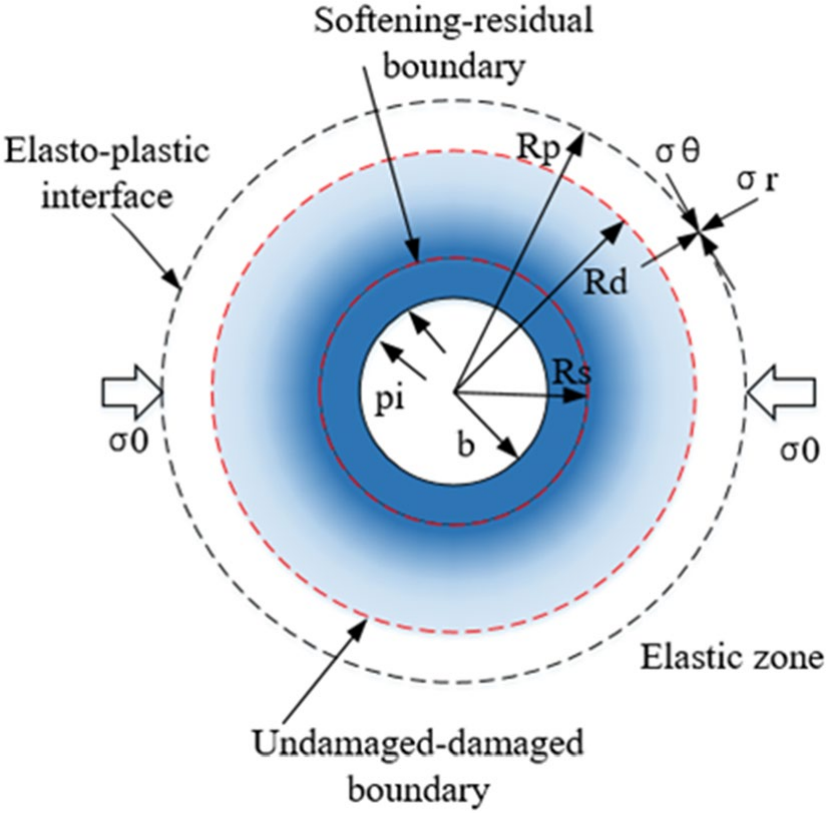
Formation of plastic and damage zones around the circular opening of a tunnel.

Currently, in most geotechnical engineering construction, e.g., mining engineering and underground traffic engineering, the excavation of rock masses in deep-buried tunnels typically involves mechanical drilling or blasting^1^. This method can be applied to most deep-buried tunnel excavation projects with high economic benefits. However, an important problem caused by this method is that the surrounding rock is damaged due to the improper control of blasting impact force and the excavation damaged zone (EDZ) is formed (Fig. 1). The damaged zone is characterized by the deterioration in the intrinsic mechanical properties, such as the strength and stiffness of the rock mass, and it may produce adverse deformation and unstable conditions in the process of tunnel excavation^2,3^. Hence, to analyze the deformation of tunnel, it is necessary to study the damage evolution mechanism of the strength parameters of the surrounding rock after tunnel excavation^4,5^. Establishing a damage evolution equation would help engineers and construction designers to evaluate the damage degree and extent of surrounding rock^6–9^.

Although the drilling and blasting technologies have been significantly improved, they still has the disadvantage of causing damage to rock mass beyond the excavation surface, and a damaged zone is inevitably formed^10^. The formation of this damage zone leads to a decline in the strength and stiffness of surrounding rock mass, that is, the stability of the surrounding rock decreases. Beyond the damaged zone, the area where the rock is unaffected by the blasting impact force is called the undamaged zone. After tunnel excavation, the plastic zone formed around the tunnel and the plastic zone can be divided into undamaged and damaged zones. Namely, the damaged zone is formed, only in a portion or in the entire the whole of the plastic zone. However, in engineering practice and research, it has been challenging to determine the relationship between the mechanical properties of the rock mass and the degree of damage has always been a hot and difficult point^10–13^. The strength and stiffness of the damaged zone and the extent of the damaged zone are the key parameters related to the stability of the tunnel. Therefore, it is necessary to select an appropriate damaged range and analyze the influence of damaged zone in tunnel excavation design.

Under the condition of the hydrostatic stress field, the stress and displacement of a circular tunnel in an isotropic rock mass can be analyzed using an analytical solution (closed solution) and a numerical solution. For example, Brown^14^ modeled the radial displacement and stress of a circular tunnel. Wang^15^ found that the solution obtained by Brown et al. had a drawback in predicting the radial displacement and therefore derived an improved solution. Carranza-Torres^16^ developed a self-similarity solution for rock masses conforming to the Hoek–Brown (H–B) criterion, which is typically considered rigorous and complex in practical applications. Moreover, a self-similarity solution was provided by Alonso^17^ by solving differential equations of equilibrium. Alonso^18^ and Sharan^19,20^ proposed an analytical solution to the stresses and displacements of a tunnel excavated in H–B rock masses, however, it cannot accurately calculate the radial displacement since the elastic strain is assumed constant in the plastic zone. Lee and Pietruszczak^21^, Park et al.^22^, Guan^23^, and Zareifard^24^ presented different numerical solutions to the stresses and displacements of a circular tunnel excavated in strain-softening rock masses. For statement the present, the vast majority literatures neglect the existence of damage zone in the rock mass when analyzing tunnel deformation^25^. For those cases, Hoke^26^ introduced a damaged factor D for the first time to characterize the damage degree of rock mass and the damaged factor D varies from zero (for undamaged zone) to one (severe fragmentation of the rock mass caused due to blasting). The damaged factor *D* can be obtained in the following two ways: by querying the table drawn by Hoke^4,26^ or by using the formula introduced by Xia^27^. Applying the damaged factor *D* to the rock mass surrounding the tunnel would significantly reduce the strength and stability of the rock mass. Therefore, the tunnel deformation calculated by considering the damaged factor *D* is closer to real project conditions. Under these conditions, there are only a few analytical and numerical solutions, that consider the limited damage and reduced strength and stiffness parameters of the rock mass. Zareifard and Fahimifar^28^ proposed a closed-form analytical solution for a circular tunnel considering the damage zone; since the range of the plastic zone is uncertain in the initial stage, the correct operation is determined by trial-and-error. Hedayat and Weems^29^ presented an analytical–numerical solution for tunnel deformation considering the damaged zone in an elastic-brittle-plastic rock mass. For strain-softening the rock mass, the semi-numerical solution for an H–B rock mass given by^3^ Zareifard and Fahimifar overestimated the displacements in the plastic zone, as it assumes a constant damage factor $D$ in the damage zone.

The objective of this study was to establish a mechanical model considering surrounding rock damage and to derive the distributions of the stress and radial displacement of a circular tunnel. To meet this objective, based on the generalized non-linear H–B criterion and strain-softening model, a numerical method for the progressive failure of rock mass in damaged zone is proposed, considering the deterioration in the strength parameters of the rock mass in the damage zone and the variation in the Young’s modulus with the damaged factor *D*.

## Problem definition and methodology

Figure 1 shows a schematic of a circular tunnel of radius *b *excavated in homogeneous and isotropic rock masses under the effect of a far-field hydrostatic stress $\sigma_{0}$. In Fig. 1, *R*_*p*_ and *R*_*d*_ represent the plastic and damage radii; $\sigma_{\theta }$ and $\sigma_{r}$ represent the tangential and radial stresses around the tunnel, respectively. In such cases, with a decrease in the internal support pressure *p*_*i*_, an elastic deformation of the surrounding rock can occur. When the support pressure *p*_*i*_ is less than the critical pressure *p*_*ic*_, a plastic zone may be formed around the tunnel opening^22^. With the formation of the plastic zone, an excavation damage zone with a radius *R*_*d*_ will gradually form inside the plastic zone^25,30^, and the damaged factor *D* gradually increases from the depth of the damage zone, reaching maximum at the critical point in the residual zone. The list of symbols used are shown in the following Table 1.

**Table 1:** List of symbols used in this article.

Notation			
$D$	Damage factor	$u$	Radial displacement
$E_{0}$	Intact rock deformation modulus	$\varphi$	Dilation angle
$v$	Poison’s ratio of the rock mass	$\rho$	Normalized radii
$GSI_{p}^{u}$	Peak geological strength index in the undamaged zone	$\rho^{d}$	Normalized damage radii
$GSI_{r}^{u}$	Residual geological strength index in the undamaged zone	$\Delta \varepsilon_{r}^{e}$	Radial elastic strain increment
$GSI_{p}^{d}$	Peak geological strength index in the damaged zone	$\Delta \varepsilon_{\theta }^{e}$	Tangential elastic strain increment
$GSI_{r}^{d}$	Residual geological strength index in the damaged zone	$\Delta \varepsilon_{r}^{p}$	Radial plastic strain increment
$R_{d}$	Damage radius	$\Delta \varepsilon_{\theta }^{p}$	Tangential plastic strain increment
$R_{p}$	Plastic radius	$\varepsilon_{\theta }^{p}$	Tangential plastic strain
$R_{s}$	Residual radius	$\varepsilon_{r}^{p}$	Radial elastic strain
$b$	Radius of tunnel	$\varepsilon_{r}$	Radial strain
$k$	Coefficient of dilation	$\varepsilon_{\theta }$	Tangential strain
$p_{i}$	Internal supporting pressure	$\sigma_{c}$	Uniaxial compressive strength
$p_{ic}$	Critical supporting pressure	$\sigma_{0}$	Hydrostatic stress
$\omega_{p}^{u}$	Peak parameter in the undamaged zone	$\sigma_{r}$	Radial stress
$\omega_{r}^{u}$	Residual parameter in the undamaged zone	$\sigma_{\theta }$	Circumferential stress
$\omega_{p}^{d}$	Peak parameter in the damaged zone	$\eta^{p}$	Softening parameter
$\omega_{r}^{d}$	Residual parameter in the damaged zone	$\eta^{p*}$	Critical deviatoric plastic strain

### Yield criterion and strain-softening behavior of materials

The strength-weakening behavior can be modeled according to the theory of plastic mechanics, which can help derive the elastoplastic deformation process of the surrounding rock^31^. Based on this theory, both the failure criterion *F* and the plastic potential function *G* depend on the stress state and the softening parameter $\eta^{p}$ of the rock mass. Therefore, it is important to select appropriate softening parameters and yield criteria^32^. In this study, the plastic shear strain $\eta^{p}$ was used as the deviator plastic strain parameter^18^:

1 $$\eta^{p} = \varepsilon_{\theta }^{p} - \varepsilon_{r}^{p}$$

where $\varepsilon_{\theta }^{p}$ and $\varepsilon_{r}^{p}$ are the tangential and radial strains representing the major and minor principal plastic strains $\varepsilon_{1}^{p}$ and $\varepsilon_{3}^{p}$, respectively. Therefore, Eq. (1) can be rewritten as follows:

2 $$\eta^{{\text{p}}} = \varepsilon_{1}^{p} - \varepsilon_{3}^{p}$$

The yield criterion can be expressed as follows:

3 $$F\left( {\sigma_{\theta } ,\;\sigma_{r} ,\;\eta^{p} } \right) = \sigma_{\theta } - \sigma_{r} - H\left( {\sigma_{r} ,\;\eta^{p} } \right)$$

The nonlinear H–B instability criterion is similar to that in the case of rock plastic deformation. Therefore, $H( {\sigma_{r} ,\;\eta^{p} } )$ can be expressed as^30^:

4 $$H\left( {\sigma_{r} ,\;\eta^{p} } \right) = \sigma_{c} \left( {\eta^{p} } \right)\left( {m\left( {\eta^{p} } \right)\frac{{\sigma_{r} }}{{\sigma_{r} \left( {\eta^{p} } \right)}} + s\left( {\eta^{p} } \right)} \right)^{{\alpha \left( {\eta^{p} } \right)}}$$

where $\sigma_{c}$ represents the uniaxial compressive strength of the rock mass; $m$, $s$, and $\alpha$ represent the strength parameters of the H–B rock mass, which can be obtained using the geological strength index (GSI)^33^. Therefore, the peak and residual values of the strength parameters can be expressed as^26^:

5 $$m_{p} = m_{i} \exp \left( {\frac{{GSI_{p} - 100}}{28 - 14D}} \right)$$

6 $$s_{p} = \exp \left( {\frac{{GSI_{p} }}{9 - 3D}} \right)$$

7 $$\alpha_{p} = 0.5 + {{\left( {\exp \left( { - \frac{{GSI_{p} }}{15}} \right) - \exp \left( { - \frac{20}{3}} \right)} \right)} \mathord{\left/ {\vphantom {{\left( {\exp \left( { - \frac{{GSI_{p} }}{15}} \right) - \exp \left( { - \frac{20}{3}} \right)} \right)} 6}} \right. \kern-\nulldelimiterspace} 6}$$

8 $$m_{r} = m_{i} \exp \left( {\frac{{GSI_{r} - 100}}{28 - 14D}} \right)$$

9 $$s_{r} = \exp \left( {\frac{{GSI_{r} }}{9 - 3D}} \right)$$

10 $$\alpha_{r} = 0.5 + {{\left( {\exp \left( { - \frac{{GSI_{r} }}{15}} \right) - \exp \left( { - \frac{20}{3}} \right)} \right)} \mathord{\left/ {\vphantom {{\left( {\exp \left( { - \frac{{GSI_{r} }}{15}} \right) - \exp \left( { - \frac{20}{3}} \right)} \right)} 6}} \right. \kern-\nulldelimiterspace} 6}$$

where the subscripts “*p*” and “*r*” denote the peak and residual values of the surrounding rock parameters *m*, *s*, $\alpha$ and *GSI*, respectively. In addition, *m*_*i*_ and *D* represent the material constant of the rock under intact conditions and the degree of rock mass damage, respectively. The residual GSI_r_ of the rock mass can be calculated using the following formula^34^.

11 $$GSI_{r} = GSI_{p} \exp \left( { - 0.0134GSI_{p} } \right)$$

In addition to estimating the *GSI* of the undamaged and damaged rock masses, estimating the deformation modulus of the rock mass is an important part of the deformation calculation. Therefore, the displacement analysis of the tunnel also requires estimating the deformation modulus of the excavated rock mass. Many scholars have shown that the deformations modulus is not a constant^35^. Based on the database of rock mass deformation modulus measurements, the following expression has been proposed for estimating the rock mass modulus^4^:

12 $$E_{\left( i \right)} = E_{0} \left( {0.02 + \frac{{1 - {D \mathord{\left/ {\vphantom {D 2}} \right. \kern-\nulldelimiterspace} 2}}}{{1 + \exp \left( {{{60 + 15D - GSI} \mathord{\left/ {\vphantom {{60 + 15D - GSI} {11}}} \right. \kern-\nulldelimiterspace} {11}}} \right)}}} \right)$$

where *E*_0_ is the intact rock deformation modulus.

### Plastic potential function of the material

Selecting an appropriate plastic potential function has an important influence on the calculation of the plastic strain process. In geo-mechanics, the Mohr–Coulomb type of plastic potential function is widely applied and implemented, which can be written as^25^:

13 $$G\left( {\sigma_{\theta } ,\;\sigma_{r} ,\;\eta^{p} } \right) = \sigma_{\theta } - k\left( {\eta^{p} } \right)\sigma_{r}$$

where $k\left( {\eta^{p} } \right)$ is the coefficient of dilation and is computed using Eq. (14).

14 $$k = \frac{1 + \sin \left( \varphi \right)}{{1 - \sin \left( \varphi \right)}}$$

where $\varphi$ is the expansion angle of the material^17^. In this study, the increment relationship between the radial and tangential plastic strains can be obtained according to the non-associated flow rule^21^.

15 $$\Delta \varepsilon_{r}^{p} = - k\left( {\eta^{p} } \right)\Delta \varepsilon_{\theta }^{p}$$

### Evolution of material parameters in different zones

**Figure 2:**
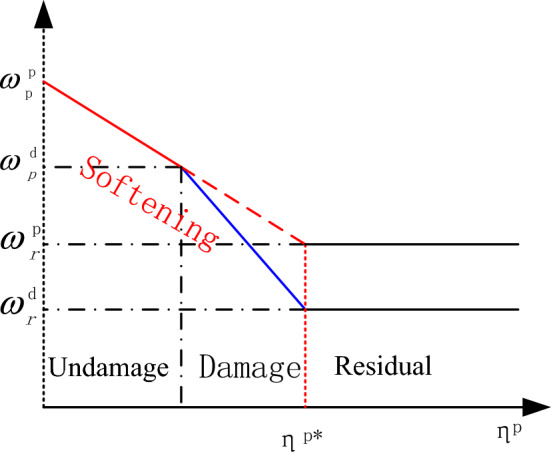
Evolution of strength parameters of the surrounding rock in the plastic zone.

Based on field experience, a new elastoplastic damage piecewise curve strain softening behavior model is established for undamaged and damaged rock masses, as shown in Fig. 2. The stress and strain behavior follows the piecewise linear softening behavior in the plastic undamaged and damaged zones, which are represented by solid red and blue lines in Fig. 2, respectively.

As mentioned above, the strength parameters of the material vary depending on the softening functions employed for the different zones. The evolution of the strength parameter of the rock mass can be described using the piecewise functions of the deviatoric plastic strain $\eta^{p}$ in the plastic undamaged and damaged zones, and calculated using Eqs. (16) and (17), respectively.

16 $$\omega^{u} \left( \eta \right) = \left\{ {\begin{array}{*{20}l} {\omega_{p}^{u} - \left( {\omega_{p}^{u} - \omega_{r}^{u} } \right)\left( {\frac{\eta }{{\eta^{*} }}} \right),\quad 0 < \eta < \eta^{*} } \\ {\omega_{r} ,\quad \eta > \eta^{*} } \\ \end{array} } \right.$$

17 $$\omega^{d} \left( \eta \right) = \left\{ {\begin{array}{*{20}l} {\omega_{p}^{d} - \left( {\omega_{p}^{d} - \omega_{r}^{d} } \right)\left( {\frac{{\eta^{d} }}{{\eta^{*} }}} \right),\quad 0 < \eta^{d} < \eta^{*} } \\ {\omega_{r}^{d} ,\quad \eta^{d} > \eta^{*} } \\ \end{array} } \right.$$

where $\omega^{u} ( \eta )$ represents one of the rock mass parameters,$m( \eta )$, $s( \eta )$, $\alpha ( \eta )$, $GSI( \eta )$ and $\varphi ( \eta )$ in the undamaged zone and $\omega^{d} ( {\eta^{d} } )$ represents one of the rock mass parameters, $m_{d} ( \eta )$, $s^{d} ( \eta )$, $\alpha^{d} ( \eta )$, $GSI^{d} ( \eta )$, $D( \eta )$ and $\varphi^{d} ( \eta )$ in the damaged zone^25^. In the above formula, $D_{p} = 0$ represents the boundary at the beginning of the damaged zone, and $D_{r}$ represents the maximum damage degree of the damage zone. $\eta^{*}$ is the critical plastic shear strain representing the starting point of the residual behavior and needs to be determined experimentally^32^. The subscripts “*p*” and “*r*” indicate the peak and residual values^21^, respectively, and the superscripts “*u*” and “*d*” represent the undamaged and damaged zones, respectively.

## Basic numerical formulations for strain-softening model

### Preliminaries

**Figure 3:**
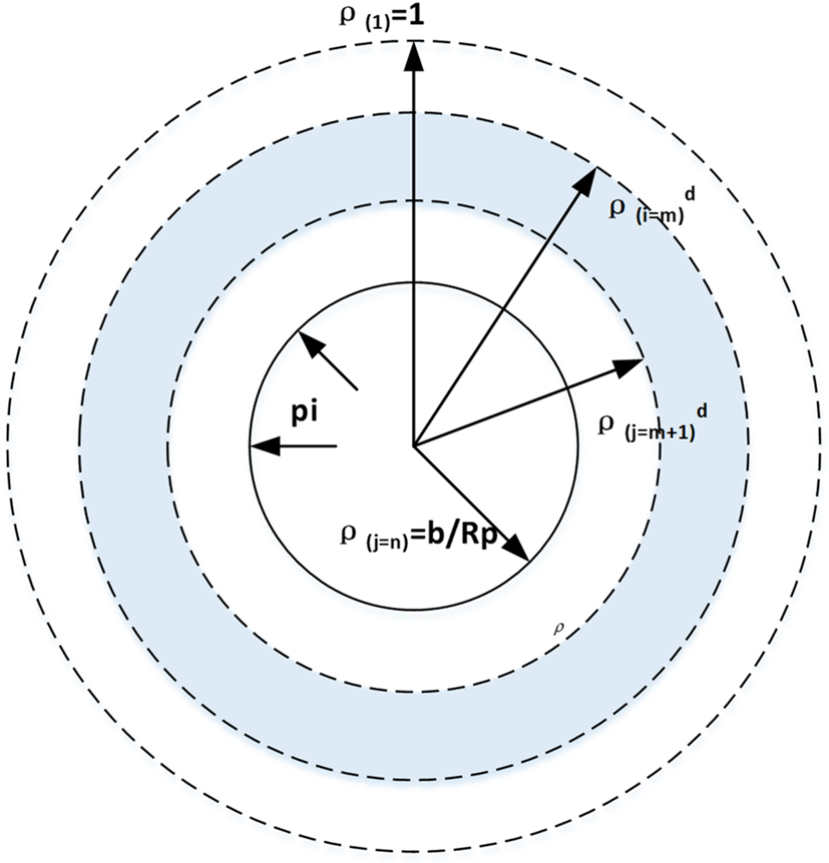
Normalized radius of the plastic zone divided into *n* annuli.

In this section, the complex stress–strain relationship is derived based on the newly defined medium. Evidently, the radial stress $\sigma_{r} = \sigma_{R}$ at $r = R_{p}$ (outer boundary of the plastic zone) in Fig. 1. Assuming that the plastic zone is composed of *n* concentric annuli, as shown in Fig. 3, the ith annulus is surrounded by two circles of normalized radii $\rho_{{( {i - 1} )}} = {{r_{{( {i - 1} )}} } \mathord{\left/ {\vphantom {{r_{{( {i - 1} )}} } {R_{p} }}} \right. \kern-\nulldelimiterspace} {R_{p} }}$ and $\rho_{( i )} = {{r_{( i )} } \mathord{\left/ {\vphantom {{r_{( i )} } {R_{{\text{p}}} }}} \right. \kern-\nulldelimiterspace} {R_{{\text{p}}} }}$ When the damaged zone is formed (${\text{r}} = R_{d}$), the normalized radius of the damage zone is $\rho^{d} = {{R_{d} } \mathord{\left/ {\vphantom {{R_{d} } {R_{p} }}} \right. \kern-\nulldelimiterspace} {R_{p} }}$.

At the elastoplastic boundary, where *i* = 1, according to the theory of elasticity, the stress and strain can be obtained as follows^14^:

18 $$\left( {\begin{array}{*{20}l} {\sigma_{r\left( 1 \right)} } \\ {\sigma_{\theta \left( 1 \right)} } \\ \end{array} } \right) = \left( {\begin{array}{*{20}l} {\sigma_{R} } \\ {2\sigma_{0} - \sigma_{R} } \\ \end{array} } \right)$$

19 $$\left( {\begin{array}{*{20}l} {\varepsilon_{r\left( 1 \right)} } \\ {\varepsilon_{\theta \left( 1 \right)} } \\ \end{array} } \right) = \frac{1 + v}{{E_{\left( 1 \right)} }}\left( {\begin{array}{*{20}l} {\sigma_{R} - \sigma_{0} } \\ {\sigma_{0} - \sigma_{R} } \\ \end{array} } \right)$$

where $\sigma$ and $\varepsilon$ represent the stress and strain, respectively, and the subscripts “*r*” and “$\theta$” represent the radial and circumferential directions, respectively. $E_{( 1 )}$ is the Young’s modulus, and $v$ is the Poisson’s ratio of the intact rock mass at the elastoplastic boundary^3^.

Based on previous research^21,32,36,37^, the radial stress increment can be expressed as follows:

20 $$\Delta \sigma_{r} = \frac{{p_{i} - \sigma_{R} }}{n}$$

where $p_{i}$ is known, and $\sigma_{R}$ is the elastoplastic critical radial stress^21^.

Thus, the radial stress components for the ith radius may be written as^21^

21 $$\sigma_{\theta \left( i \right)} = \sigma_{{r\left( {i - 1} \right)}} + \Delta \sigma_{r}$$

According to the H–B yield criterion, the corresponding hoop stress can be expressed as:

22 $$\sigma_{\theta \left( i \right)} = \sigma_{r\left( i \right)} + H\left( {\sigma_{r\left( i \right)} ,\eta_{{\left( {i - 1} \right)}}^{p} } \right)$$

where *H* is defined in Eq. (4).

23 $$\Delta \sigma_{\theta \left( i \right)} = \sigma_{\theta \left( i \right)} - \sigma_{{\theta \left( {i - 1} \right)}}$$

For the rock mass in the plastic zone, the elastic strain in terms of the stress considering the initial hydrostatic stress $\sigma_{0}$, can be expressed as:

24 $$\left( {\begin{array}{*{20}l} {\varepsilon_{r\left( i \right)}^{e} } \\ {\varepsilon_{\theta \left( i \right)}^{e} } \\ \end{array} } \right) = \frac{1 + v}{E}\left[ {\begin{array}{*{20}l} {1 - v} & { - v} \\ { - v} & {1 - v} \\ \end{array} } \right]\left( {\begin{array}{*{20}l} {\sigma_{{r\left( {\text{i}} \right)}} - \sigma_{0} } \\ {\sigma_{0} - \sigma_{\theta \left( i \right)} } \\ \end{array} } \right)$$

Based on the plane strain condition, the elastic strain increment is calculated according to Hooke’s law using the radial and tangential stress increments as follows ^32^:

25 $$\left( {\begin{array}{*{20}l} {\Delta \varepsilon_{r\left( i \right)}^{e} } \\ {\Delta \varepsilon_{\theta \left( i \right)}^{e} } \\ \end{array} } \right) = \frac{1 + v}{E}\left[ {\begin{array}{*{20}l} {1 - v} & { - v} \\ { - v} & {1 - v} \\ \end{array} } \right]\left( {\begin{array}{*{20}l} {\Delta \sigma_{r\left( i \right)} } \\ {\Delta \sigma_{\theta \left( i \right)} } \\ \end{array} } \right)$$

Note that E_(*i*)_ varies with the GSI and damage factor $D$ and can be obtained using Eq. (12).

### Basic numerical formulations for the plastic undamaged zone

Under plane strain and axisymmetric conditions, the equilibrium equation and coordination equation under polar coordinates can be expressed using the normalized radius $\rho_{\left( i \right)} = {{r_{\left( i \right)} } \mathord{\left/ {\vphantom {{r_{\left( i \right)} } {R_{p} }}} \right. \kern-\nulldelimiterspace} {R_{p} }}$:

26 $$\frac{{d\sigma_{r} }}{dr} + \frac{{\sigma_{r} - \sigma_{\theta } }}{r} = 0$$

or

27 $$\frac{{d\varepsilon_{\theta } }}{dr} + \frac{{\varepsilon_{\theta } - \varepsilon_{r} }}{r} = 0$$

Approximately, for the ith annulus, Eq. (26) (can be transformed into:

28 $$\frac{{\sigma_{r\left( i \right)} - \sigma_{{r\left( {i - 1} \right)}} }}{{\rho_{\left( i \right)} - \rho_{{\left( {i - 1} \right)}} }} + \frac{{2H\left( {\overline{\sigma }_{r} ,\eta_{{\left( {i - 1} \right)}} } \right)}}{{\rho_{\left( i \right)} + \rho_{{\left( {i - 1} \right)}} }} = 0$$

where $\overline{\sigma }_{r} = {{( {\sigma_{( i )} + \sigma_{{( {i - 1} )}} } )} \mathord{\left/ {\vphantom {{( {\sigma_{( i )} + \sigma_{{( {i - 1} )}} } )} 2}} \right. \kern-\nulldelimiterspace} 2}$. Therefore, the normalized radius $\rho_{( i )}$ can be written as:

29 $$\rho_{\left( i \right)} = \frac{{2H\left( {\overline{\sigma }_{r} ,\eta_{{\left( {i - 1} \right)}} } \right) + \Delta \sigma_{r} }}{{2H\left( {\overline{\sigma }_{r} ,\eta_{{\left( {i - 1} \right)}} } \right) - \Delta \sigma }}\rho_{{\left( {i - 1} \right)}}$$

For the undamaged zone, the geometric equations in a polar coordinate system and the total strains of each annulus can be decomposed into elastic and plastic parts as follows^38^:

30 $$\begin{array}{*{20}l} {\varepsilon_{r} = \frac{du}{{dr}}} & {\varepsilon_{\theta } = \frac{u}{r}} \\ \end{array}$$

31 $$\left( {\begin{array}{*{20}l} {\varepsilon_{r} } \\ {\varepsilon_{\theta } } \\ \end{array} } \right) = \left( {\begin{array}{*{20}l} {\varepsilon_{r}^{e} } \\ {\varepsilon_{\theta }^{e} } \\ \end{array} } \right) + \left( {\begin{array}{*{20}l} {\varepsilon_{r}^{p} } \\ {\varepsilon_{\theta }^{p} } \\ \end{array} } \right)$$

Therefore, the compatibility Eq. (27) can be expressed as

32 $$\frac{{d\varepsilon_{\theta }^{p} }}{d\rho } + \frac{{\varepsilon_{\theta }^{p} - \varepsilon_{r}^{p} }}{\rho } = - \frac{{d\varepsilon_{\theta }^{e} }}{d\rho } - \frac{{\varepsilon_{\theta }^{e} - \varepsilon_{r}^{e} }}{\rho }$$

or

33 $$\frac{{\varepsilon_{\theta \left( i \right)}^{p} - \varepsilon_{{\theta \left( {i - 1} \right)}}^{p} }}{{\rho_{\left( i \right)} - \rho_{{\left( {i - 1} \right)}} }} + \frac{{\varepsilon_{\theta \left( i \right)}^{p} - \varepsilon_{r\left( i \right)}^{p} + \varepsilon_{{\theta \left( {i - 1} \right)}}^{p} - \varepsilon_{{r\left( {i - 1} \right)}}^{p} }}{{\rho_{\left( i \right)} + \rho_{{\left( {i - 1} \right)}} }} = - \frac{{\Delta \varepsilon_{\theta \left( i \right)}^{e} }}{{\Delta \rho_{\left( i \right)} }} - \frac{1 + v}{{E_{\left( i \right)} }}\left( {\frac{{2H\left( {\overline{\sigma }_{r\left( i \right)} ,\eta_{{\left( {i - 1} \right)}}^{p} } \right)}}{{\rho_{\left( i \right)} + \rho_{{\left( {i - 1} \right)}} }}} \right)$$

34 $$A_{{\left( {\text{i}} \right)}} = - \frac{{\Delta \varepsilon_{\theta \left( i \right)}^{e} }}{{\Delta \rho_{\left( i \right)} }} - \frac{1 + v}{{E_{\left( i \right)} }}\left( {\frac{{2H\left( {\overline{\sigma }_{r\left( i \right)} ,\eta_{{\left( {i - 1} \right)}}^{p} } \right)}}{{\rho_{\left( i \right)} + \rho_{{\left( {i - 1} \right)}} }}} \right)$$

Using the difference equation, Eq. (15) can be written as:

35 $$\left\{ {\begin{array}{*{20}l} {\varepsilon_{r\left( i \right)}^{p} = \varepsilon_{{r\left( {i - 1} \right)}}^{p} - k_{{\left( {i - 1} \right)}} \left( {\varepsilon_{\theta \left( i \right)}^{p} - \varepsilon_{{\theta \left( {i - 1} \right)}}^{p} } \right)} \\ {\varepsilon_{\theta \left( i \right)}^{p} = \left( {\varepsilon_{r\left( i \right)}^{p} - \varepsilon_{{r\left( {i - 1} \right)}}^{p} } \right)/ (- k_{{\left( {i - 1} \right)}}) + \varepsilon_{{\theta \left( {i - 1} \right)}}^{p} } \\ \end{array} } \right.$$

Substituting Eq. (15) into Eq. (33), yields the following formula:

36 $$\begin{gathered} \varepsilon_{r\left( i \right)}^{p} = \frac{{ - 2k_{{\left( {{\text{i}} - 1} \right)}} A_{\left( i \right)} \Delta \rho_{\left( i \right)} \overline{\rho }_{\left( i \right)} + \varepsilon_{{\theta \left( {i - 1} \right)}}^{p} \left( {2k_{{\left( {i - 1} \right)}}^{2} \rho_{{\left( {i - 1} \right)}} + 2k_{{\psi \left( {i - 1} \right)}} \Delta \rho_{\left( i \right)} } \right)}}{{\Delta \rho_{\left( i \right)} - 2k_{{\left( {i - 1} \right)}} \rho_{{\left( {i - 1} \right)}} }} + \hfill \\ \frac{{\varepsilon_{{r\left( {i - 1} \right)}}^{p} \left( {\Delta \rho_{\left( i \right)} - 2k_{{_{{\left( {i - 1} \right)}} }} \Delta \rho_{\left( i \right)} - 2\rho_{{\left( {i - 1} \right)}} k_{{\left( {i - 1} \right)}} } \right) - 2k_{{\left( {i - 1} \right)}}^{2} \rho_{{\left( {i - 1} \right)}} }}{{\Delta \rho_{\left( i \right)} - 2k_{{\left( {i - 1} \right)}} \rho_{{\left( {i - 1} \right)}} }} \hfill \\ \end{gathered}$$

37 $$\varepsilon_{\theta \left( i \right)}^{p} = \frac{{2A_{\left( i \right)} \Delta \rho_{\left( i \right)} \overline{\rho }_{\left( i \right)} - \varepsilon_{{\theta \left( {i - 1} \right)}}^{p} \left( {2k_{{\left( {i - 1} \right)}} \rho_{{\left( {i - 1} \right)}} - \Delta \rho_{\left( i \right)} } \right) + 2\varepsilon_{{r\left( {i - 1} \right)}}^{p} \Delta \rho_{{\left( {i - 1} \right)}} }}{{\Delta \rho_{\left( i \right)} - 2k_{{\left( {i - 1} \right)}} \rho_{{\left( {i - 1} \right)}} }}$$

where $$\Delta \rho_{ ( i )} = \rho_{ ( i )} - \rho_{{ ( {i - 1} )}}$$.

In the above equation, the dilation angle $\varphi ( {\eta^{p} } )$ varies with the increase in the softening parameter $\eta^{p}$, and $k_{{( {i - 1} )}}$ varies with the dilation angle. The deviatoric plastic shear strain $\eta_{( i )}^{p}$ at the ith annulus can be updated as follows:

38 $$\eta_{\left( i \right)}^{p} = \varepsilon_{\theta \left( i \right)}^{p} - \varepsilon_{r\left( i \right)}^{p}$$

Finally, the total radial and hoop strain at the ith annulus can be obtained as follows:

39 $$\left\{ {\begin{array}{*{20}l} {\varepsilon_{r\left( i \right)} = \varepsilon_{r\left( i \right)}^{p} + \varepsilon_{r\left( i \right)}^{e} } \\ {\varepsilon_{\theta \left( i \right)} = \varepsilon_{\theta \left( i \right)}^{p} + \varepsilon_{\theta \left( i \right)}^{e} } \\ \end{array} } \right.$$

### Calculation of the stress–strain relationship in the damaged zone

In this study, we consider that the plastic region is divided into undamaged and damaged zones, as shown in Fig. 2, and assume that the stress and strain behaviors of the rock mass are continuous in the plastic zone. The solid red and blue lines represent the rock mass evolution functions in the undamaged and damaged zones, respectively. When the above-described process is repeated *m* times, i.e., $\rho_{{( {i = m} )}} = \rho^{d}$. Subsequently, the surrounding rock is transformed from an undamaged zone to a damaged zone.

For the damage zone, the yield criterion can be expressed as follows^30^:

40 $$\sigma_{\theta } - \sigma_{r} = H^{d} \left( {\sigma_{r} ,\eta^{d} } \right)$$

As introduced above, because of the excavation-induced effects, the material parameters vary with the softening parameter and damage factor *D* in the damaged zone. For the H–B yield criterion, $H^{d} ( {\sigma_{r} ,\eta } )$ can be expressed as:

41 $$H^{d} \left( {\sigma_{r} ,\eta } \right) = \sigma_{c} \left( \eta \right)\left( {m^{d} \left( \eta \right)\frac{{\sigma_{r} }}{{\sigma_{c} \left( \eta \right)}} + s^{d} \left( \eta \right)} \right)^{{\alpha^{d} \left( \eta \right)}}$$

where $m^{d}$,$s^{d}$, and $\alpha^{d}$ are the strength parameters of the H–B criterion in the damaged zone, which can be calculated using $GSI$ through Eqs. (42), (43), and (44).

42 $$m_{p}^{d} = m_{i} \exp \left( {\frac{{GSI_{p}^{d} - 100}}{28 - 14D}} \right)$$

43 $$s_{p}^{d} = \exp \left( {\frac{{GSI_{p}^{d} - 100}}{9 - 3D}} \right)$$

44 $$\alpha_{p}^{d} = 0.5 + {{\left( {\exp \left( {\frac{{GSI_{p}^{d} }}{15}} \right) - \exp \left( { - \frac{20}{3}} \right)} \right)} \mathord{\left/ {\vphantom {{\left( {\exp \left( {\frac{{GSI_{p}^{d} }}{15}} \right) - \exp \left( { - \frac{20}{3}} \right)} \right)} 6}} \right. \kern-\nulldelimiterspace} 6}$$

The residual values of m, s, and ${\upalpha }$ in the damaged zone can be rewritten as:

45 $$m_{r}^{d} = m_{i} \exp \left( {\frac{{GSI_{r}^{d} - 100}}{28 - 14D}} \right)$$

46 $$s_{r}^{d} = \exp \left( {\frac{{GSI_{r}^{d} - 100}}{9 - 3D}} \right)$$

47 $$\alpha_{r}^{d} = 0.5 + {{\left( {\exp \left( {\frac{{GSI_{r}^{d} }}{15}} \right) - \exp \left( { - \frac{20}{3}} \right)} \right)} \mathord{\left/ {\vphantom {{\left( {\exp \left( {\frac{{GSI_{r}^{d} }}{15}} \right) - \exp \left( { - \frac{20}{3}} \right)} \right)} 6}} \right. \kern-\nulldelimiterspace} 6}$$

where the subscripts “*p*” and “*r*” denote the peak and residual values of the material parameters in the damaged zone. When $\rho_{{ ( {{\text{i}} = m} )}} = \rho^{d}$, $GSI_{p}^{d} = GSI_{{ ( {i = m} )}}$ can be obtained, and $GSI_{r}^{d}$ can be calculated using Eq. (11).

As mentioned above, the stress and strain are continuous at the critical point in the damaged and undamaged zones. Under this condition, $i = m$ represents the last annulus of the undamaged zone, and $j ( {m \le j \le n} )$ represents the number of annuli in the excavation damaged zone^25^. Thus, the radial stress for the jth annulus can be written as:

48 $$\sigma_{r\left( j \right)} = \sigma_{{r\left( {j - 1} \right)}} + \Delta \sigma_{r}$$

and the hoop stress can be expressed as:

49 $$\sigma_{\theta \left( j \right)} = \sigma_{r\left( j \right)} + H^{d} \left( {\sigma_{r\left( j \right)} ,\eta_{{\left( {j - 1} \right)}}^{p} } \right)$$

Thus, $$\Delta \sigma_{\theta } = \sigma_{\theta ( j )} - \sigma_{{\theta_{{( {j - 1} )}} }}$$.

The elastic strain increments in the radial and tangential directions in the damaged zone can be obtained using Eq. (24), and the Young’s modulus is replaced with a new Young’s modulus $E_{( j )}^{d}$.

The governing equilibrium equations for the damaged zone are different from those for the undamaged zone given that the damage of the surrounding rock is considered. Therefore, the equilibrium equation and coordination equations under polar coordinates can be expressed as

50 $$\frac{{d\sigma_{r} }}{{d\rho^{d} }} + \frac{{H^{d} \left( {\sigma_{r} ,\eta } \right)}}{{\rho^{d} }} = 0$$

51 $$\frac{{d\varepsilon_{\theta } }}{{d\rho^{d} }} + \frac{{\varepsilon_{\theta } - \varepsilon_{r} }}{{\rho^{d} }} = 0$$

Hence, Eq. (50) can be approximated for the jth annulus as follows:

52 $$\frac{{\sigma_{r\left( j \right)} - \sigma_{{r\left( {j - 1} \right)}} }}{{\rho_{\left( j \right)} - \rho_{{\left( {j - 1} \right)}} }} + \frac{{2H^{d} \left( {\overline{\sigma }_{r} ,\eta_{{\left( {j - 1} \right)}} } \right)}}{{\rho_{\left( j \right)}^{d} + \rho_{{\left( {j - 1} \right)}}^{d} }} = 0$$

where $\overline{\sigma }_{r ( j )} = {{ ( {\sigma_{r ( j )} + \sigma_{{r ( {j - 1} )}} } )} \mathord{\left/ {\vphantom {{( {\sigma_{r ( j )} + \sigma_{{r( {j - 1} )}} } )} 2}} \right. \kern-\nulldelimiterspace} 2}$, and the normalized radius $\rho_{( j )}^{d}$ can be expressed as follows:

53 $$\rho_{\left( j \right)}^{d} = \frac{{2H^{d} \left( {\overline{\sigma }_{r} ,\eta_{{\left( {j - 1} \right)}} } \right) + \Delta \sigma_{r} }}{{2H^{d} \left( {\overline{\sigma }_{r} ,\eta_{{\left( {j - 1} \right)}} } \right) - \Delta \sigma }}\rho_{{\left( {j - 1} \right)}}^{d}$$

Because the strain is composed of elastic and plastic parts, Eq. (51) can be reformulated as:

54 $$\frac{{\varepsilon_{\theta \left( j \right)}^{p} - \varepsilon_{{\theta \left( {j - 1} \right)}}^{p} }}{{\rho_{\left( j \right)} - \rho_{{\left( {j - 1} \right)}} }} + \frac{{\varepsilon_{\theta \left( j \right)}^{p} - \varepsilon_{r\left( j \right)}^{p} + \varepsilon_{{\theta \left( {j - 1} \right)}}^{p} - \varepsilon_{{r\left( {j - 1} \right)}}^{p} }}{{\rho_{\left( j \right)} + \rho_{{\left( {j - 1} \right)}} }} = A_{\left( j \right)}$$

where $$A_{( j )} = - \frac{{\Delta \varepsilon_{\theta ( j )}^{e} }}{{\Delta \rho_{( j )} }} - \frac{1 + v}{{E_{( j )} }}( {\frac{{2H^{d} ( {\overline{\sigma }_{r( j )} ,\eta_{{( {j - 1} )}} } )}}{{\rho_{( j )}^{d} + \rho_{{( {j - 1} )}}^{d} }}} )$$.

Similar to Eqs. (36) and (37), the radial plastic strain and the circumferential plastic strain in the damage zone can be obtained:

55 $$\begin{gathered} \varepsilon_{r\left( j \right)}^{p} = \frac{{ - 2k_{{\left( {j - 1} \right)}} A_{\left( j \right)} \Delta \rho_{\left( j \right)}^{d} \overline{\rho }_{\left( j \right)} + \varepsilon_{{\theta \left( {j - 1} \right)}}^{p} \left( {2k_{{\left( {j - 1} \right)}}^{2} \rho_{{\left( {j - 1} \right)}}^{d} + 2k_{{\left( {i - 1} \right)}} \Delta \rho_{\left( j \right)}^{d} } \right)}}{{\Delta \rho_{\left( j \right)}^{d} - 2k_{{\left( {i - 1} \right)}} \rho_{{\left( {j - 1} \right)}}^{d} }} + \hfill \\ \frac{{\varepsilon_{{r\left( {j - 1} \right)}}^{p} \left( {\Delta \rho_{\left( j \right)}^{d} - 2k_{{_{{\left( {j - 1} \right)}} }} \Delta \rho_{\left( j \right)}^{d} - 2\rho_{{\left( {j - 1} \right)}}^{d} k_{{\left( {j - 1} \right)}} } \right) - 2k_{{\left( {i - 1} \right)}}^{2} \rho_{{\left( {j - 1} \right)}}^{d} }}{{\Delta \rho_{\left( j \right)}^{d} - 2k_{{\left( {i - 1} \right)}} \rho_{{\left( {j - 1} \right)}}^{d} }} \hfill \\ \end{gathered}$$

56 $$\varepsilon_{\theta \left( j \right)}^{p} = \frac{{2A_{\left( j \right)} \Delta \rho_{\left( j \right)}^{d} \overline{\rho }_{\left( j \right)} - \varepsilon_{{\theta \left( {j - 1} \right)}}^{p} \left( {2k_{{\left( {j - 1} \right)}} \rho_{{\left( {j - 1} \right)}}^{d} - \Delta \rho_{\left( j \right)}^{d} } \right) + 2\varepsilon_{{r\left( {j - 1} \right)}}^{p} \Delta \rho_{\left( j \right)}^{d} }}{{\Delta \rho_{\left( j \right)} - 2k_{{\left( {j - 1} \right)}} \rho_{{\left( {j - 1} \right)}} }}$$

In Eqs. (55) and (56), $\Delta {\rho }_{(j)}^{d}={\rho }_{(j)}^{d}-{\rho }_{(j-1)}^{d}$. Subsequently, the plastic shear strain $\upeta$ can be expressed as:

57 $$\eta_{\left( j \right)} = \varepsilon_{\theta \left( j \right)}^{p} - \varepsilon_{r\left( j \right)}^{p}$$

Hence, similar to Eq. (39), the total radial and tangential strains can be obtained:

58 $$\left\{ {\begin{array}{*{20}l} {\varepsilon_{r\left( j \right)} = \varepsilon_{r\left( j \right)}^{p} + \varepsilon_{r\left( j \right)}^{e} } \\ {\varepsilon_{\theta \left( j \right)} = \varepsilon_{\theta \left( j \right)}^{p} + \varepsilon_{\theta \left( j \right)}^{e} } \\ \end{array} } \right.$$

The normalized displacement can be determined using Eq. (56).

59 $$U_{\left( j \right)} = \varepsilon_{\theta \left( j \right)} \rho_{\left( j \right)}$$

When the above calculation is iterated n times, the radial displacement of the tunnel surrounding rock is calculated as follows:

60 $$u_{\left( j \right)} = U_{\left( j \right)} R_{p}$$

## Verification and comparison

To prove the accuracy of the proposed method, which was implemented in MATLAB, the results obtained using the proposed solution are compared with those obtained by Mohammad Reza Zareifard^3^ and Lee and Pietruszczak^21^.

The rock strength parameters corresponding to Zareifard’s^3^ study were taken as input data: b = 7 m, $\sigma_{0} = 27\;MPa$, $p_{i} = 5.14\;MPa$, $\sigma_{c} = 90\;MPa$, $m_{i} = 10$,$E_{0} = 50\;MPa$, $v = 0.25$ (where $E_{0}$ is the Young’s modulus of the intact rock). These data were used to derive the strength parameters of the rock masses in the different zones.

### Model verification

The accuracy of the algorithm can be verified by comparing its results with the existing results in literature without considering the disturbance damage of the surrounding rock.

**Figure 4:**
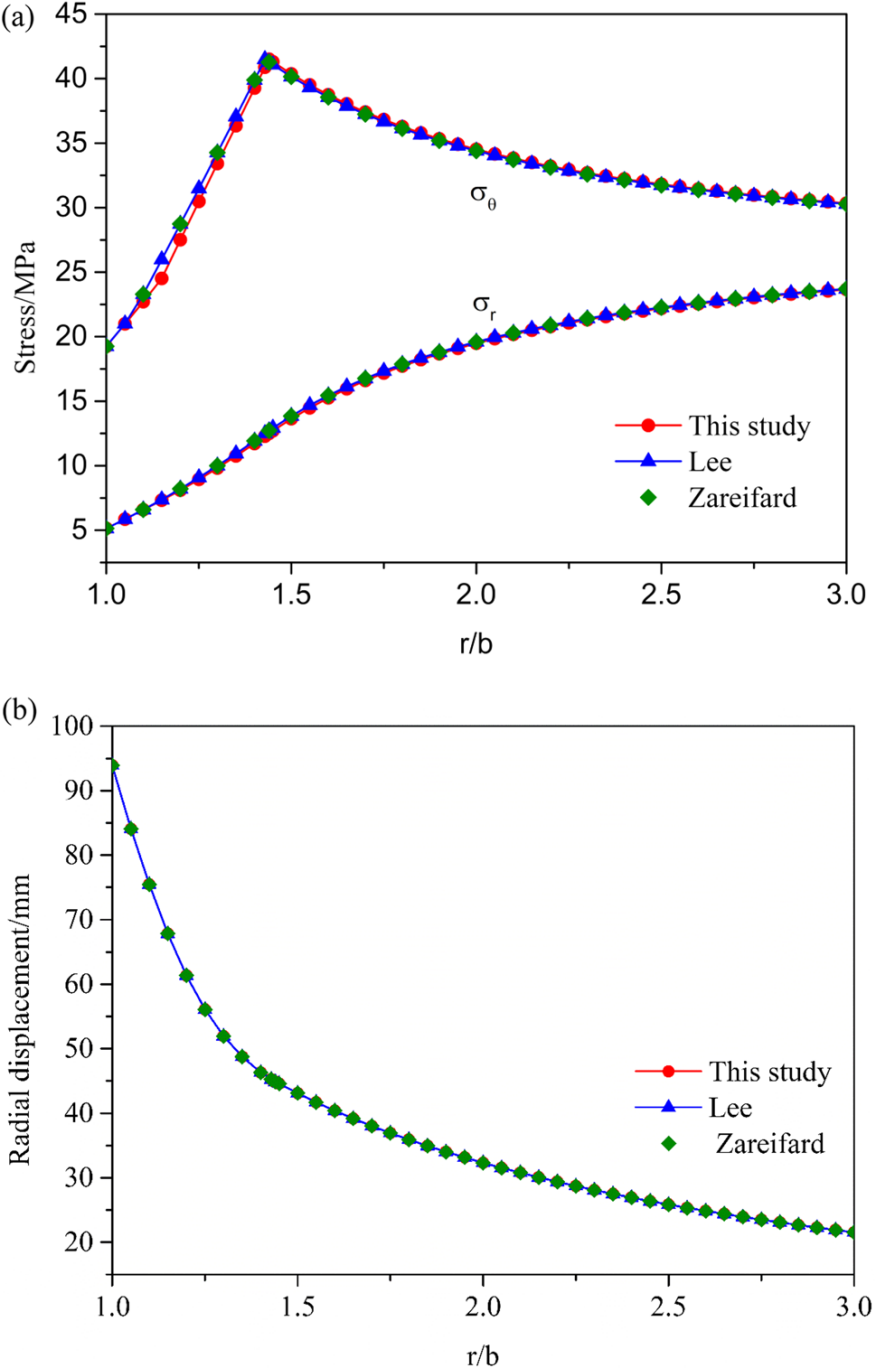
Comparison between two previous methods (Lee and Pietruszczak 2008 and Zareifard 2020) in terms of their elastoplastic solutions to the radial and circumferential stresses and radial displacements: (**a**) Radial and hoop stress, (**b**) Radial displacement.

The formation of the damaged zone around the tunnel is not considered, and hence, no excavation damage-related formula was used for comparison. Figure 4a,b present the results of the two compared methods, which yield the same results as those obtained using the proposed algorithm, thus confirming the accuracy of the new user-coded algorithm.

### Comparison with Lee and Pietruszczak’s solutions

To show the effectiveness of the proposed algorithm, the problem originally solved by Lee and Pietruszczak^21^ was solved using the proposed method and then compared. The values of the damage factor *D*_*r*_ were set to 0.2 and 0.4, and the results were compared with those obtained by Lee and Pietruszczak, who did not consider the damaged zone formation.

**Figure 5:**
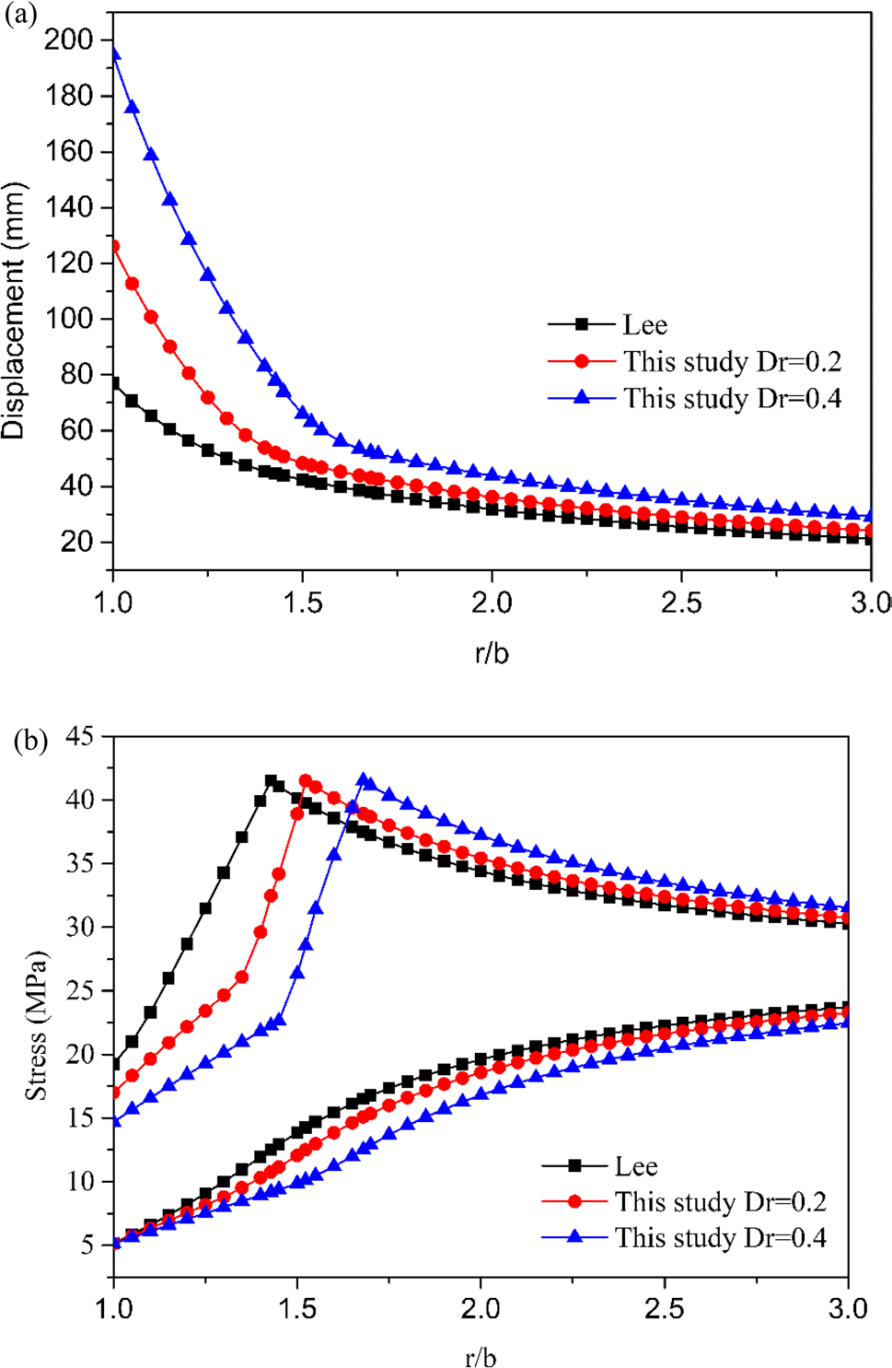
(**a**) Distribution of radial and hoop stresses, (**b**) Distribution of radial displacement.

Figure 5 shows the variation curves of the stress and radial displacement versus *r*/*b* after tunnel excavation. As shown, for *D*_*r*_ ranging from 0 to 0.4, the radial displacement increases whereas the tangential stress decreases at the tunnel excavation surface.

### Comparison with the results obtained by Zareifard and Fahimifar

A semi-numerical solution for an axisymmetric circular tunnel excavated in a damaged rock mass is obtained using the algorithm proposed by Zareifard and Fahimifar^3^. The solution was proposed for solving circular tunnel excavation problems with a nonlinear H–B failure criterion.

**Figure 6:**
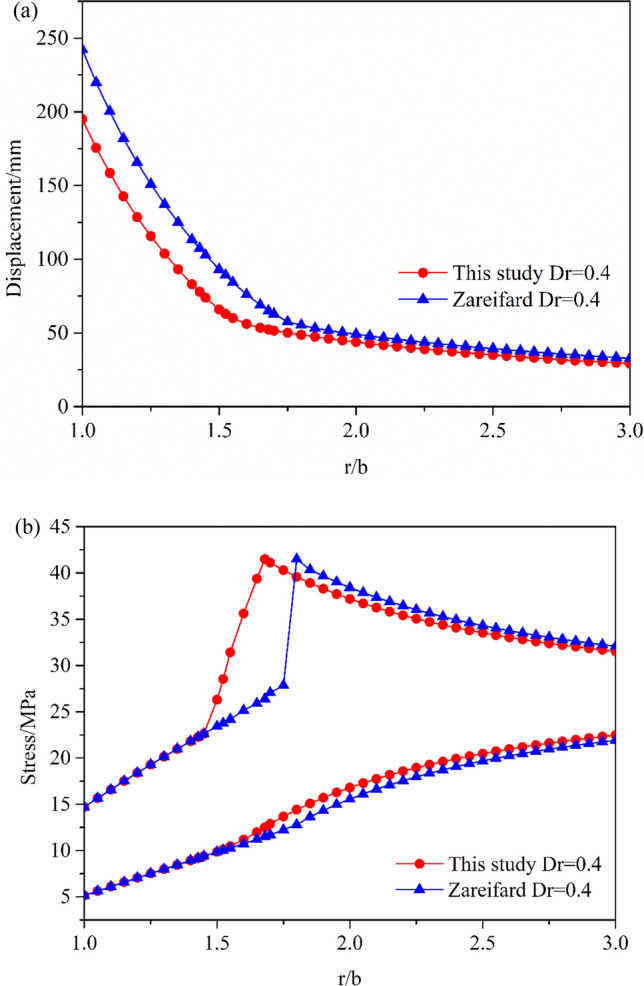
Comparison with the results reported by Zareifard and Fahimifar: (**a**) Radial and circumferential stresses; (**b**) Radial displacement.

Figure 6 shows the distributions of the stresses and radial displacement calculated using both the approaches. Figure 6a shows that the distributions of $\sigma_{\theta }$ and $\sigma_{r}$ are largely the same in the plastic residual zone, because the damage factor *D*_*r*_ of the algorithm is equal to the damaged factor *D* of Zareifard and Fahimifar’s algorithm for the residual zone. In Zareifard and Fahimifar’s algorithm, the damage factor *D* is a constant value for the damaged zone, which can lead to sudden changes in the circumferential stress at the critical points in the elastoplastic zone. However, the authors proposed that the damaged factor *D* changes gradually from the elastoplastic critical point $D_{p} = 0$ to the residual zone *D*_*r*_; therefore, the $\sigma_{\theta }$ distribution shows evident differences in the plastic zone. Meanwhile, the displacement calculated by Zareifard and Fahimifar is higher that calculated in this study.

## Influence of new variables on strain-softening behavior

To illustrate the influence of the new variables on the results of the algorithm, the influence of each variable in the algorithm is studied in separate sections. In this case, the damage factor *D* and dimensionless damage radius $\rho^{d}$ are the main parameters characterizing the degree and extent of damage.

### Effect of gradual variation damaged factor D

The parameter *D*_*r*_ represents the maximum disturbance degree of the surrounding rock. Four common *D*_*r*_ values, 0, 0.2, 0.4, and 0.6, were used to investigate the role of the damage factor *D* in the stress distribution and ground response curve (GRC).

**Figure 7:**
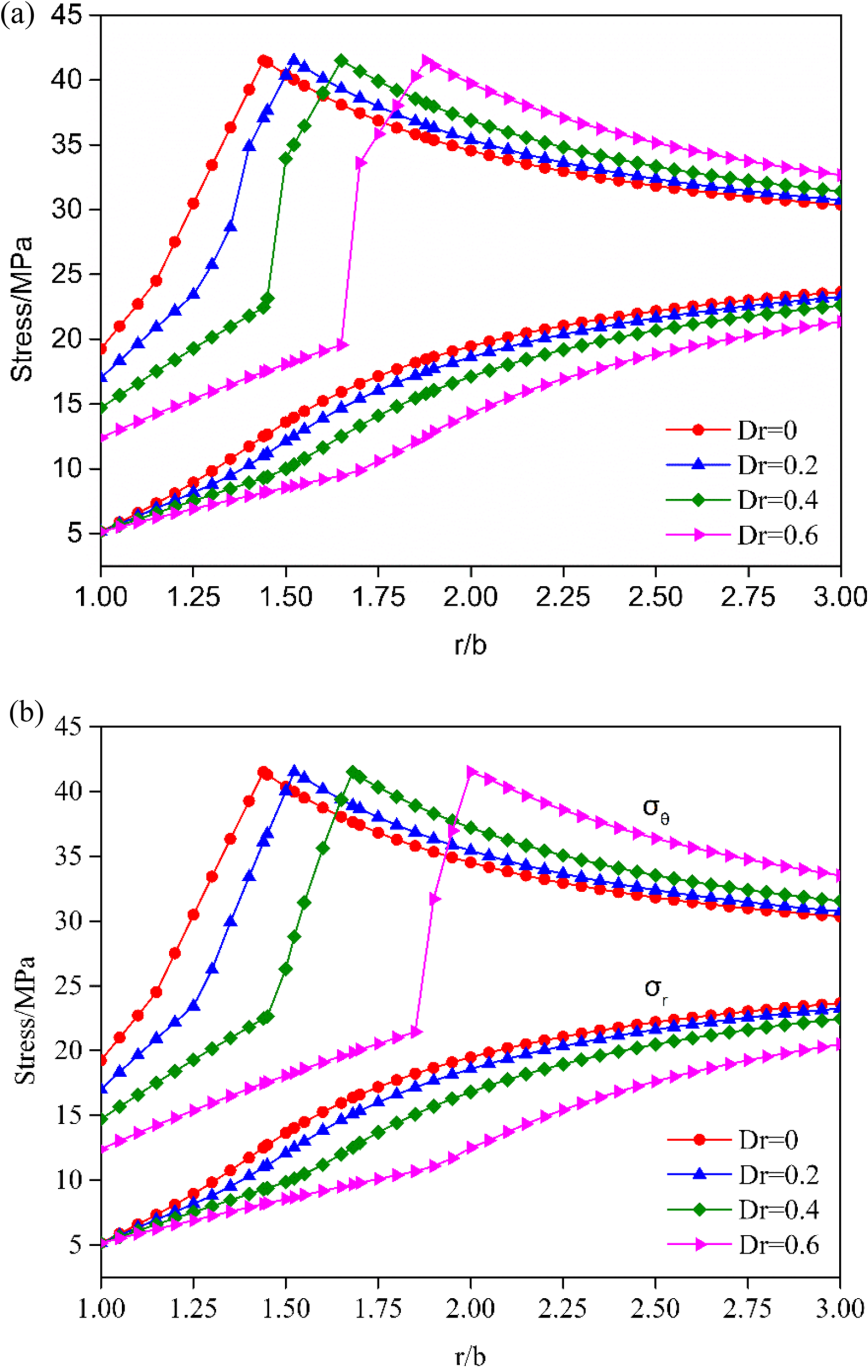
Distributions of radial and circumferential stresses for elastic-strain-softening behavior under different damage factors *D*: (**a**) (${\uprho }^{d} = 1$), (**b**) (${\uprho }^{d} = 0.9$).

Figure 7a,b show the variation curves of the stress versus *r*/*b* in the elastoplastic zone for $\rho^{d} = 1$ and $\rho^{d} = 0.9$. It is apparent that the circumferential stress increases gradually with *r*/*b* and reaches maximum at the elastoplastic boundary. As shown in Fig. 7, with the increase in the damaged factor *D*_*r*_, the circumferential stresses decrease on the surface of the tunnel opening, the inflection point between the plastic softening zone and the plastic residual zone is more evident, and the range of the plastic residual region increases significantly. As the rock mass damage occurs in the plastic zone (Fig. 7b), the circumferential stress shifts at the critical point between the damaged and undamaged zones. In addition, there is a remarkable drop in the distribution of $\sigma_{\theta }$ in the damaged zone at higher damage factor values, which means that the increasing rate of the circumferential stress increases with the increase in the damage factor *D*_*r*_. Meanwhile, with the increase in *D*_*r*_, the slope of the radial stress curves decreases in the residual zone but increases in the softening area.

**Figure 8:**
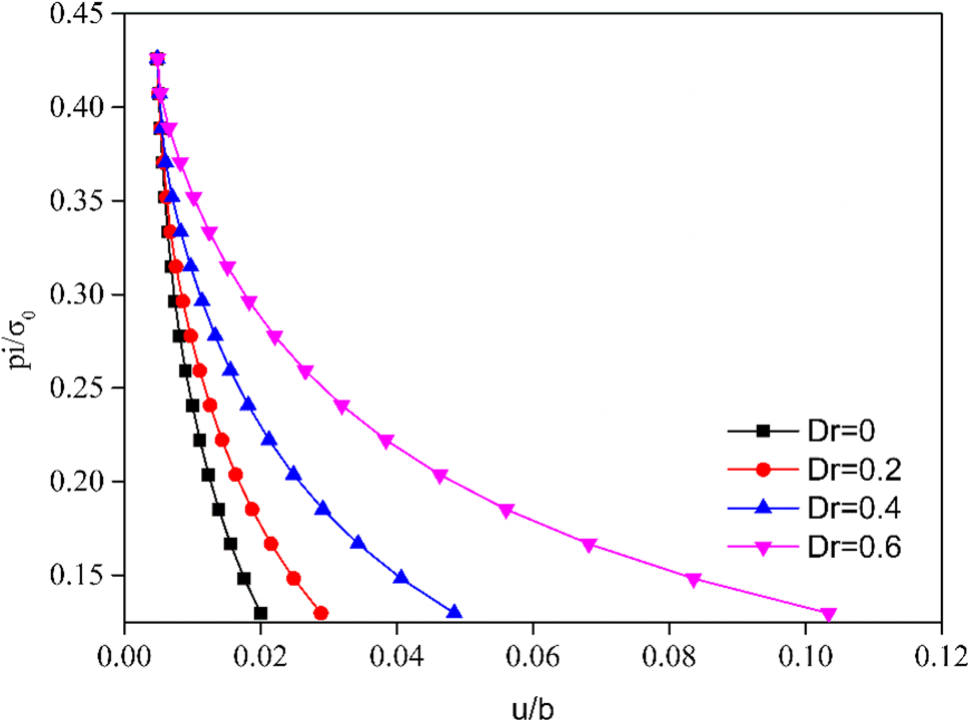
Evolution of plastic radius of strain-softened H–B rock mass with different damage factors *D*.

**Figure 9:**
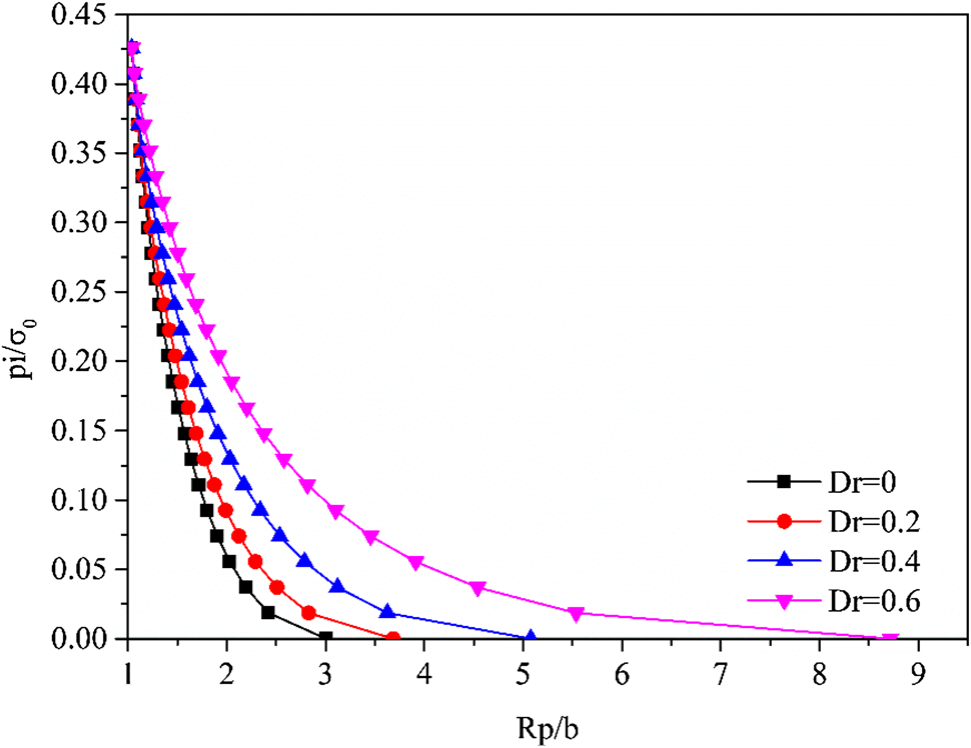
Ground reaction curve for a strain-softening H–B rock mass with different damage factors *D*.

The evolution of the plastic radius *R*_*p*_ versus the support pressure *p*_*i*_ is plotted in Fig. 8, where a distinct difference in the evolution of the plastic radius can be observed for different damage factors *D*_*r*_. For different damage factors *D*_*r*_, the plastic radius *R*_*p*_ calculated under the same $\rho^{d}$ condition increases with the decrease in the support pressure. Additionally, the plastic radius *R*_*p*_ increased with the increase of damaged factor *D*_*r*_ from 0 to 0.6. The GRC more intuitively presents the inverse relationship between the support pressure *p*_*i*_ and the radial displacement at the tunnel excavation surface. Figure 9 depicts the GRC for different damaged factors *D*_*r*_. With the increase in the damage factor *D*_*r*_ or the decrease in the support pressure, the radial displacement at the excavation boundary gradually increases. Furthermore, the effects of the damaged factor *D*_*r*_ on the radial displacement are evident, particularly when the damage factor is $D_{r} = 0.6$.

### Influence of damaged radius

As previously mentioned, the normalized damage radius $\rho^{d}$ represents the range of the surrounding rock disturbed by blasting or excavation. To investigate the influences of $\rho^{d}$ on the stress and GRC, we selected three different radii $\rho^{d}$ = 0.95, 0.9, and 0.85, and the damaged degree of the rock mass was set to 0.4.

**Figure 10:**
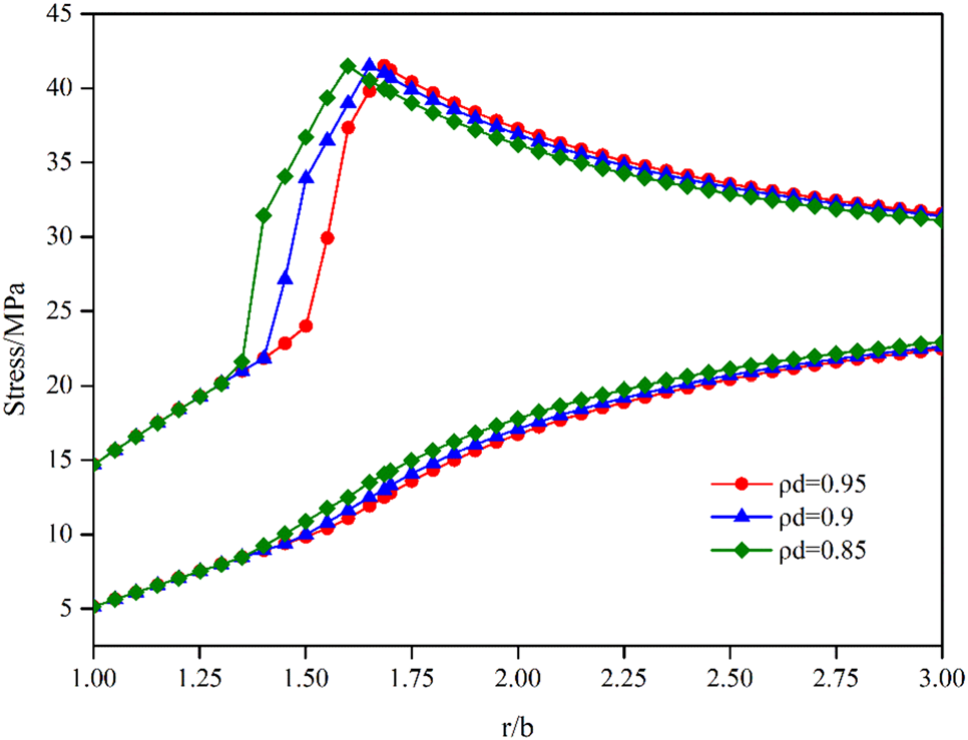
Distributions of radial and tangential stresses for elastic-strain-softening behavior under different values of damage radius ${\uprho }^{d}$.

Figure 10 shows the distributions of the hoop stress $\sigma_{\theta }$ and radial stress $\sigma_{r}$ with different normalized damage radii $\rho^{d}$ in the plastic region. As shown, the distribution of $\sigma_{\theta }$ is largely the same in the residual zone, whereas it shows evident difference under different normalized damage radii $\rho^{d}$ in the softening zone. However, with the change in the damaged radius, the distribution of $\sigma_{r}$ shows no significant difference, and the tangential stress can be divided into two distinct stages: an undamaged zone stage and a damaged zone stage in the plastic-softening process. Thus, for $\rho^{d}$ ranging from 0.95 to 0.85, the transition points of the distribution of $\sigma_{\theta }$ from the plastic-softening zone to the residual zone and the undamaged zone to the damaged zone are evidently shifted to the left. However, the drop modulus of the circumferential stress increases with the decrease in the normalized damage radius in the damage zone.

**Figure 11:**
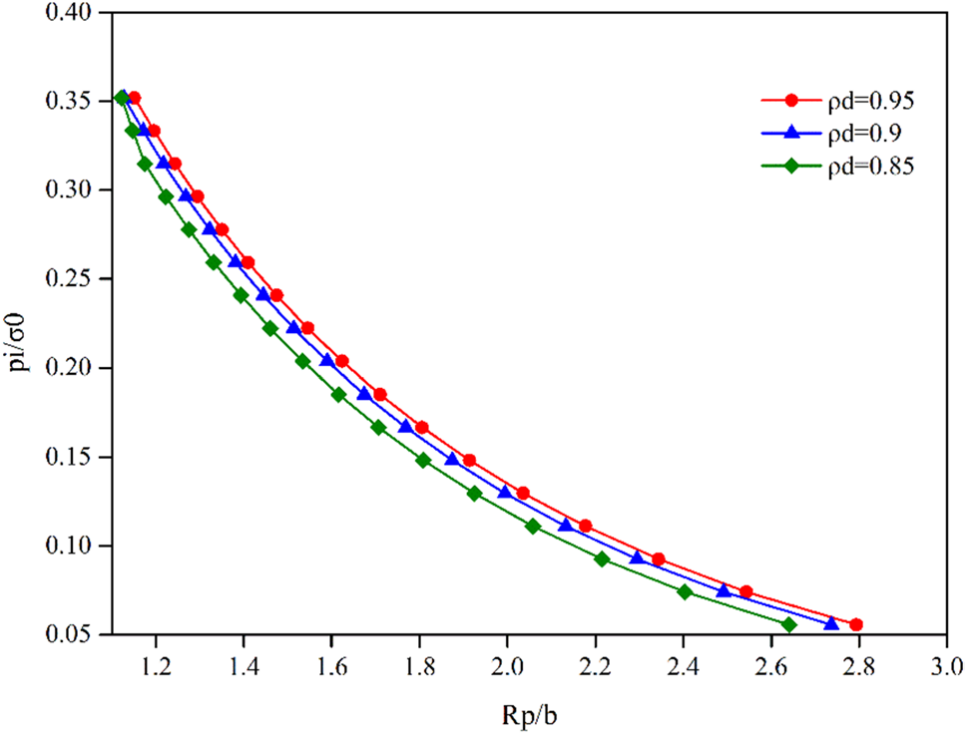
Influence of normalized damage radius ${\uprho }^{d}$ on the analysis of plastic radius ${\text{R}}_{p}$ under different internal pressures.

**Figure 12:**
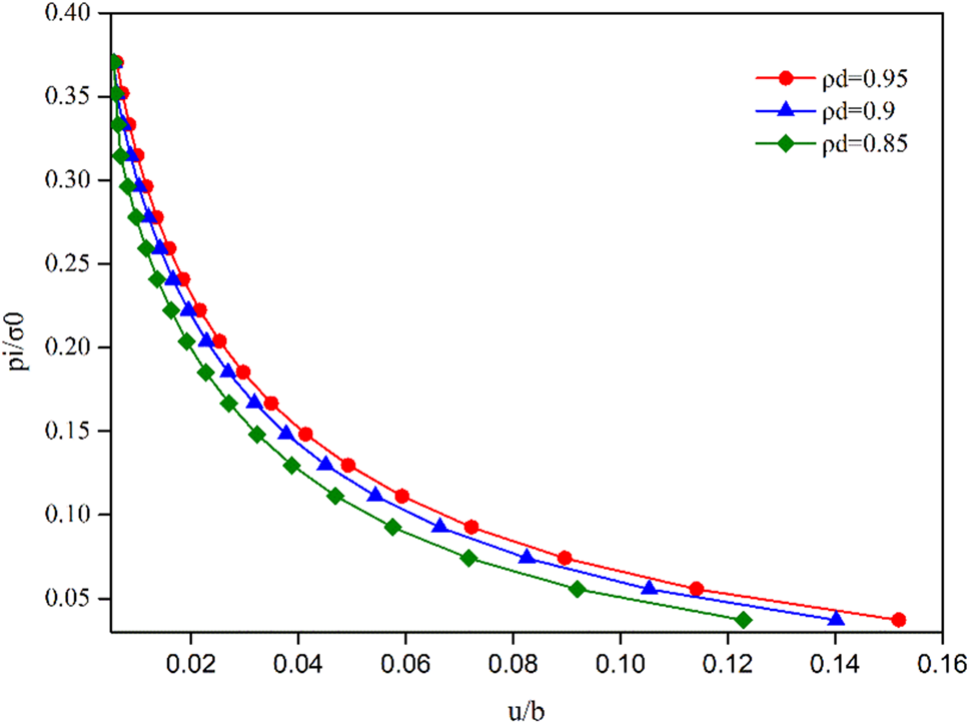
Influence of normalized damage radius ${\uprho }^{d}$ on the analysis of GRC under different internal pressures.

The evolutions of the plastic radius *R*_*p*_ and the radial displacement of the tunnel surface versus the support pressure are plotted in Figs. 11 and 12. Both these figures demonstrate a distinct difference in the evolution of the plastic radius and the GRC for different normalized damage radius $\rho^{d}$. They reveal that the evolutions of the normalized damage radius have some influence on the plastic radius curve and GRC. With the decrease in $\rho^{d}$, the plastic radius curve and GRC shift to the left, i.e., the radial displacements and plastic radius decrease under all internal support pressures.

## Conclusions

In this study, the plastic zone comprises undamaged and damaged zones, where the damaged zone represents a gradual transition from a softening zone to a plastic residual zone. Based on the theory of progressive failure, a numerical solution for a circular tunnel excavated in a strain-softening rock mass was developed. In the finite extent damage zone, the reduced strength and stiffness of the surrounding rock with the gradual change in the damage factor *D* is considered for more accurate numerical results. The accuracy of the proposed algorithm, which takes advantage of two solution schemes (the iterative finite difference solution proposed by Lee and Pietruszczak and consideration of the damage zone as done by Zareifard 2020), was verified through some examples. The following conclusions can be drawn from the study:

1. By simplifying the algorithm as a special case, the results were found to be in good agreement with those of other methods. The algorithm could accurately reflect the strain-softening problem of a circular tunnel excavated in a rock mass.
2. While solving for the stress–strain states in the plastic zone, the variation in the Young’s modulus proposed by Hoek, gradual variation in the damage factor $D$ and normalized damage radius $\rho^{d}$ were considered, and the obtained results were compared with those of previous algorithms. Without considering the damaged factor during surrounding rock excavation, Lee’s result found to be relatively conservative, and the damaged factor $D$ in the damaged zone was constant, leading to an overestimation of the calculation result reported by Zareifard.
3. The comparative results showed that the variation in the damaged factor has distinct effects on the evolutions of $\sigma_{\theta }$, $\sigma_{r}$, $R_{p}$, and GRC in the plastic zone. The normalized damaged radius $\rho^{d}$ slightly affected the evolutions of $\sigma_{\theta }$, $\sigma_{r}$, $R_{p}$, and GRC.

In a nutshell: selecting an appropriate model that considers the damage radius and the variation in the damaged factor $D$ is of great significance for analyzing the GRC of circular tunnels in rock masses exhibiting strain-softening behavior.
